# National Medical Convention

**Published:** 1840-01-25

**Authors:** 


### NATIONAL MEDICAL CONVENTION.

The National Medical Convention for the revision of the Pharmacopœia of the United States, assembled in the City Hall, Washington, on the 1st day of January, 1840.

The following delegates represented their respective Medical Societies and Colleges in the Convention, viz.:

*Rhode Island Medical Society*—Theophilus C. Dunn, M. D.

*New Jersey Medical Society*—Lewis Condict, M. D.

*The College of Physicians of Philadelphia*—Franklin Bache, M. D., Henry Bond, M. D., Joseph Carson, M. D.

*University of Pennsylvania*—Geo. B. Wood, M. D.

*Jefferson Medical College*—Robley Dunglison, M. D.

*Delaware Medical Society*—William B. Morris, M. D., James Couper, M. D.

*Washington University, Baltimore*—John R. W. Dunbar, M. D., John C. S. Monkur, M. D., Edward Foreman, M. D.

*Medical and Chirurgical Faculty of Maryland*—Joshua J. Cohen, M. D.

*Medical Society of the District of Columbia*—Thomas Sewall, M. D., N. W. Worthington, M. D.

*Columbian Medical College*—Thomas Miller, M. D., Harvey Lindsly, M. D., John W. Thomas, M. D.

*Vincennes Medical Society of Indiana*—John W. Davis, M. D.

*Georgia Medical Society*—William Bacon Stevens, M. D.

The credentials of the delegates from the White Mountains Medical Society of Vermont, from the Medical Society of New Hampshire, from the Albany Medical College, and from the College of Physicians and Surgeons of Lexington, Kentucky, were presented by Dr. Condict, President of the Convention of 1830; but the delegates were prevented from attending. After the rising of the Convention, however, Josiah Bartlett, M. D., delegate from the New Hampshire Medical Society, and Samuel G. Baker, M. D., and William A. Aiken, M. D., delegates from the University of Maryland, reached Washington; and, by public notice in the papers, stated their full concurrence in the measures adopted by the Convention.

The Convention elected Lewis Condict, M. D., of New Jersey, President; George B. Wood, M. D., of Philadelphia, Vice President; N. W. Worthington, M. D., of Georgetown, D. C., Secretary; and Harvey Lindsly, M. D., of Washington, Assistant Secretary.

With the view of giving the various medical interests of the country their due weight in the deliberations of the Convention, the Surgeon General of the Army, and the Senior Naval Surgeon at Washington, were invited to participate in the proceedings.

After some other preliminary business, the Convention adopted the following resolution, offered by Dr. Bache:

*Resolved*, That the delegates from the different medical bodies represented in this Convention, be requested to present any written communications with which they may have been charged.

Upon calling over the several delegations, it appeared that no written communication had been forwarded to the Convention, except by the College of Physicians of Philadelphia. Dr. Bache presented from this College several documents, which he stated had been prepared with great industry and care, with a view to facilitate the revision and emendation of the Pharmacopœia of 1830. This communication elicited discussion; but with a view to more definite action, Dr. Lindsly proposed the following resolution, which was adopted.

*Resolved*, That the communication from the College of Physicians of Philadelphia be referred to a committee, who shall also be instructed to report a plan by which the revision and publication of the Pharmacopœia may be carried into effect.

It was resolved that the committee should consist of five members, to be named by the President; and Drs. Bache, Davis, Stevens, Cohen, and Dunn, were appointed.

Dr. Wood offered the following proposition, which was adopted.

*Resolved*, That a committee be appointed to report a plan for the organization of the next Convention for revising the Pharmacopœia.

It was ordered that the committee consist of three members, to be named by the President; and Drs. Wood, Sewall, and Dunglison, were appointed.

The committee to whom the documents from the College of Physicians of Philadelphia were referred, and whose duty it was to arrange a plan by which the revision and publication of the Pharmacopœia might be carried into effect, made the following report, which, with the accompanying resolution, was adopted by the Convention:

"The committee are of opinion, that the labours of revision, constituting the communication from the College of Physicians, would form a proper basis for the new Pharmacopœia; and that this communication and all others that shall be received from bodies which have appointed delegates to this Convention, should be referred to a committee of revision and publication to meet in Philadelphia as soon as practicable. As it is desirable that the Committee here proposed should have the assistance of Pharmaceutical bodies, it is recommended that authority be given to it to request the co-operation of Colleges of Pharmacy in the United States. A revising Committee thus constituted, and clothed with power to fill their own vacancies, to publish the work after the completion of the revision, and to take order on all incidental measures necessary to carry out the objects of the Convention, would, in the opinion of this Committee, form a body, to which the revision and publication of the Pharmacopœia might be safely trusted. To carry out these views, the Committee recommend the adoption of the following resolutions by the Convention:

"1. The communication from the College of Physicians of Philadelphia, and all other communications which may be received from bodies that have appointed delegates to this Convention, shall be referred to a Committee of revision and publication, consisting of seven members, three of whom shall form a quorum.

"2. The Committee thus constituted, shall meet in Philadelphia, and be convened as soon as practicable by its chairman.

"3. The Committee shall be authorized to request the co-operation of the Colleges of Pharmacy in the United States, to publish the work after the completion of the revision, and to take all other measures which they may deem necessary to carry into effect the object of the Convention.

"4. The Committee shall have power to fill its own vacancies.

"5. When the Committee shall have terminated their labours, they shall prepare a report of their proceedings, and transmit it to the Secretary of this Convention, to be laid before the next Convention."

All which is respectfully submitted.

Franklin Bache,
Jno. W. Davis,
Wm. Bacon Stevens, } Committee.
Joshua J. Cohen,
Theophilus C. Dunn,

*Washington, Jan.* 3, 1840.

The Convention then proceeded to choose the members of the Committee of revision and publication proposed in the above report, and Drs. Wood, Bache, Dunglison, Cohen, Dunn, Stevens and Sewall, were appointed.

The committee whose duty it was to arrange a plan for the organization of the next Convention for revising the Pharmacopœia, made a report, which, at the suggestion of Dr. Stevens, was amended, so as to make the first Monday in May, 1850, the time for the meeting of the Convention, instead of the first Monday in January, 1850. The report thus amended, and modified in other respects to suit the change, was adopted by the Convention as follows:

"The Committee appointed to suggest a plan for organizing the next Convention, report, that they have taken the subject into consideration, and ask leave to submit the following resolutions, which, with a few modifications, are the same as those adopted in 1830, for the organization of the present Convention.

"1. The President of this Convention shall, on the first day of May, 1849, issue a notice, requesting the several incorporated State Medical Societies, the incorporated Medical Colleges, the incorporated Colleges of Physicians and Surgeons, and the incorporated Colleges of Pharmacy, throughout the United States, to elect a number of delegates not exceeding three, to attend a general Convention to be held at Washington, on the first Monday in May, 1850.

"2. The several incorporated bodies thus addressed shall also be requested by the President to submit the Pharmacopœia to a careful revision, and to transmit the result of their labours, through their delegates, or through any other channel, to the next Convention.

"3. The several medical and pharmaceutical bodies shall be further requested to transmit to the President of this Convention the names and residences of their respective delegates as soon as they shall have been appointed, a list of whom shall be published, under his authority, for the information of the medical public, in the newspapers and medical journals, in the month of March, 1850.

"4. In the event of the death, resignation, or inability to act of the President of the Convention, these duties shall devolve on the Vice President; and should the Vice President also be prevented from serving, upon the Secretary, or the Assistant Secretary, the latter acting in the event of the inability of the former."

George B. Wood,

Thos. Sewall, } Committee.

Robley Dunglison,

*Washington*, January 3, 1840.

The following resolutions were offered by Dr. Wood, and adopted by the Convention:

*Resolved*, 1st, That the Secretary take charge of and preserve the existing records until his successor be appointed by the Convention of 1850, when it shall be his duty to hand them over to such successor; 2d, that in case of the death, resignation, or inability to act of the Secretary, his duties shall devolve upon the Assistant Secretary; and, 3d, that it be recommended to future Conventions to appoint their Secretary and Assistant Secretary from members residing in the District of Columbia.

Dr. Bond offered the following resolution, which was adopted:

*Resolved*, That the Committee of Revision and Publication be requested to take such measures as they may deem most effective, to induce physicians and apothecaries to adopt the nomenclature of the Pharmacopœia in their prescriptions and labels.

Dr. Dunglison offered the following resolution:

*Resolved*, That the officers of this Convention be requested to prepare forthwith for publication, such part of the transactions of this Convention as may seem to them to be adapted for making extensively known its important objects and proceedings, and that they be authorized to publish the same in the various medical journals of the United States, and in such of the daily and other newspapers as they may think proper.

This resolution was adopted, and it was made the duty of the Secretary and Assistant Secretary to carry it into effect.

Having transacted business of great interest to the medical profession of their country,—having passed votes of thanks to the officers of the Convention "for the able and dignified manner in which they had discharged their respective duties," and to the Board of Aldermen of Washington for the use of their Hall,—the Convention, after a session of three days, characterized by a spirit of generous cordiality, which must contribute greatly to secure the objects for which they assembled, adjourned.

By order,

N. W. Worthington, Secretary.
Harvey Lindsly, Ass't Sec'ry.

P. S.—The medical journals throughout the United States are respectfully requested to copy the foregoing abstract of the proceedings of the Convention.

